# Inhibiting or promoting: Population aging and economic development in China

**DOI:** 10.1371/journal.pone.0303197

**Published:** 2024-05-30

**Authors:** XiFeng Yang, MeiHui Qi

**Affiliations:** 1 School of International Education, Jiangxi Science & Technology Normal University, Nanchan, Jiangxi Province, China; 2 Department of Business Administration, Lingnan Normal University, Zhanjiang, Guangdong, China; Lahore School of Economics, PAKISTAN

## Abstract

Population aging has become a social issue of concern to the whole world, and as the world’s most populous country, how to cope with population aging will be a hot issue that all sectors of Chinese society must think about. This paper uses provincial panel data from 30 provinces in China from 2000 to 2021 to study the relationship between population aging and economic development based on the perspective of health expenditure. The DIFF-GMM model, the fixed effect model (FE), and fixed effect instrumental variable model (FE-IV) are used to test this study. The following two conclusions are drawn from the empirical study: (1) population aging has a significant inhibitory effect on economic development, while health expenditures have a significant promotional effect on economic development; and (2) increased health expenditures help to alleviate the negative impact of population aging on economic development. However, the deepening of population aging will likewise inhibit the positive effect of health expenditure on economic growth. Based on the conclusions of the study, it is recommended that the government and society should continue to increase spending in the field of health protection, encourage and guide residents to carry out self-care, and moderately increase personal health expenditure, to promote economic development with healthy bodies and realize the goal of "Healthy China".

## 1. Introduction

As the global population continues to grow, population aging is gradually becoming a hot issue of global concern [1]. As the most populous country and the second largest economy in the world, China’s study of the impact of population aging on economic development is informative for other developing countries. The United Nations clearly defined in 2019 that the region is considered to have entered an aging society when the proportion of the elderly population aged 65 and above reaches 7% of the total population, and the region is considered to have entered a moderately aging society when the proportion of the elderly population aged 65 and above reaches 14% of the total population. By the end of 2022. China’s elderly population aged 65 years and over will number 209.78 million, accounting for 14.9 percent of the total population, which has already reached the United Nations standard of "moderate aging".

In addition to the economic problems caused by the rapid expansion of China’s aging population, the health of China’s aging population is also an important constraint on the further development of China’s economy [1]. In addition to the economic problems caused by the rapid expansion of China’s aging population, the health of China’s aging population is also an important constraint to further economic development. According to the results of the survey conducted by the China Office for the Aged and other relevant departments, at present, about 190 million elderly people in China are suffering from chronic diseases, more than 40 million elderly people are incapacitated or semi-incapacitated, there are more than 15 million patients with dementia, and there are as many as 29 million people with disabilities above the age of 60 years old, and the problem of elderly people’s "longevity but unhealthy" has been further highlighted with the increase in the speed of China’s aging population. The problem of the "long but unhealthy life" of the elderly has been further highlighted by the increase in the speed of China’s aging.

As China ages and the number of "disabled" and "semi-disabled" elderly people increases year by year, health expenditure will face a severe test [2]. According to the China Statistics Year 2022, health expenditure will face a severe challenge. According to the 2022 China Statistical Yearbook, the total health expenditure in 2022 will reach 768,449,900,000,000 Yuan, 466,999,000,000 Yuan higher than that in 2020. The problem of "long life but no health" of the elderly is becoming more and more prominent, and the total cost of health expenditure is increasing year after year, which also brings constraints to the sustainable development of the economy and society [3].

The China Aging Science Research Center predicts that the consumption potential of China’s elderly population may exceed 40,000 billion yuan by 2050 and that the silver-hair economy is expected to fuel further economic development and become the next growth point for China’s economic development. However, the long-standing problem of the "long and unhealthy life" of the elderly in China will inevitably hinder the development of the silver-hair economy. Therefore, how to promote economic development against the background of the increasing number of elderly people and the problem of "unhealthy longevity" is one of the key issues in the development of the elderly population. The health of the elderly population is one of the most important issues [4].

To this end, the report of the Twentieth National Congress of the Communist Party of China and the Fourteenth Five-Year Plan both point out the need to promote the construction of a healthy China, optimize the population development strategy, implement a national strategy for actively coping with the aging of the population, develop the elderly care business and the elderly care industry, and build an elderly care system that combines medical care, health care, and recreation. At the same time, we have taken measures to gradually delay the statutory retirement age and optimize the birth policy, develop the silver-hair economy, develop age-appropriate technologies and products, and cultivate new business models such as smart old-age care to actively cope with the negative factors brought by the "aging" society [5]. Although the implementation of the above policies can alleviate the negative impact of an aging society on China’s economic development for some time, over time, the population will continue to age [6,7]. However, over time, the "crowding out effect" of population aging and disablement on China’s labor force has not been fundamentally resolved [8].

In existing studies, more attention has been paid to the relationship between population aging and economic development, and fewer studies have explored the impact of population aging on economic development based on the perspective of national health expenditures. Therefore, there is no accurate conclusion as to whether national health expenditures can alleviate the impacts of population aging on economic development, and whether health expenditures and population aging will boost the silver-hair economy to become the next growth point of China’s economic development. In summary, to explore the impact of population aging on regional economic development, this paper is based on the provincial panel data of 30 provinces, municipalities, and autonomous regions in China from 2000 to 2021, and adopts the DIFF-GMM model, which incorporates population aging, regional economic development, and national health expenditures into the same research framework, and analyzes the impacts of population aging and health expenditures on China’s economic development by using empirical studies respectively. The impact of population aging and health expenditure on China’s economic development is analyzed separately using empirical studies. At the same time, the empirical study goes one step further by introducing the interaction term between health expenditure and population aging to explore the impact of population aging and health expenditure on economic development.

The three main contributions of this study are as follows:

First, China is the world’s largest developing country, and studying the impact of population aging on economic development in China provides experience for other developing countries.

Second, based on the perspective of health expenditure, we analyze the role of health expenditure in the relationship between population aging and economic development, as well as analyze the interaction between population aging and health expenditure on economic development, to contribute to the improvement of academic research.

Thirdly, China’s economic development can be said to be a global miracle, but the time to enter the aging society is also earlier than that of the generally developed countries, so there are differences in the role of the aging society on economic development in different periods, so this study will divide the impact of China’s population aging on economic development into two time periods: 2000–2010 and 2011–2021, to explore the impact of population aging on economic development in different periods and enrich the relevant academic literature.

The research structure of this paper is as follows: firstly, the relationship between population aging and economic development is presented in the introduction, and the analysis of references is carried out through problem orientation. Secondly, through the organization and analysis of the literature, the current conclusions on the relationship between population aging and economic development are drawn in the academic world. Thirdly, designing variables, screening data, designing models, and conducting empirical tests with problem orientation. Finally, according to the empirical results to get the research conclusions. At the same time, development recommendations are given based on the empirical findings.

## 2. Literature review and research hypothesis

### 2.1 Population aging and economic development

As the world’s demographic transformation accelerates, almost all countries around the globe are facing the challenges posed by population aging, and the timing and speed of this demographic transition are markedly asymmetric across countries [9]. Population structure is an important factor affecting economic development, in which population aging will have both direct and indirect impacts on the economy. With the arrival of the aging population, China’s demographic structure is rapidly changing, and the period of rapid economic development brought about by the demographic dividend has entered a bottleneck. In the existing research on the impact of population aging on the economy, it is mainly divided into the following three points of view:

First, most scholarly research has concluded that population aging leads to a slowdown in the rate of economic growth for the [10,11]. The specific principle is that population aging inhibits economic development through the reduction of labor force and productivity. The severity of population aging will have an impact on demographic change, while low fertility rates will further exacerbate this negative impact, leading to slower labor force growth and a reduction in the social labor participation rate, all of which will severely inhibit the growth of the national economy. In the studies of Maestas Nicole [12], Cylus Jonathan [13], and Jang Han-ik [14], it was found that population aging will affect the rate of economic growth, and the results of the study show that the increase in the proportion of the elderly population will have a direct correlation with the decline in GDP, which is manifested in the decline in the rate of increase in employment and labor productivity. This finding is similar to that of Ichiro Muto [15]. Besides, there are similar studies in Korea, Japan, and China. Chinese scholar Wang Wei constructs a three-phase alternating generations model with two-way intergenerational transfer to explore the impact of population aging on savings, human capital investment decisions, and economic growth of Chinese households, and the empirical results show that population aging not only slows down the rate of economic growth but also exacerbates the negative impact of the continuous decline in fertility [16]. Taking the analysis a step further, the decline in labor productivity is inevitable as age and physical functioning decline. Withdrawal from the labor force due to a lack of physical strength, knowledge structure, and innovation capacity [17] brings about the double negative impact of lower labor productivity and consumption [18]. At the same time due to the withdrawal of the elderly population from the labor force, thus leading to a decline in income, the elderly will choose to further inhibit consumption and increase personal savings, all of the above situations are not conducive to economic development [19]. In addition to the direct impacts of population aging on the economy, it has been found that population aging will bring about a reduction in the ecological footprint [20], an increase in public debt [21], and a decline in the capacity to innovate [22]. To cope with the negative impacts of an aging society on economic development, the government will increase spending on healthcare, infrastructure, pensions, etc., all of which will lead to a decrease in GDP growth [23]. The above measures will all lead to a decrease in the GDP growth rate. At the same time, population aging in developing countries can even have a significant negative impact on the country’s export upgrading [24] and will slow down economic growth as the population ages [25].

In the second view, some scholars believe that population aging will hurt the economy, and naturally, some scholars explore the positive impact of population aging on economic growth from other perspectives [26]. First of all, although population aging means a decrease in the working population, it also means an increase in regional life expectancy, economic level, and medical level. Based on the perspective of the "second demographic dividend", when the labor force just exits the labor market and enters the old age stage, there is often a higher savings situation, and because China’s pension system is more perfect, so in this period, whether it is the individual’s health or capital savings, they are still at a high level. Therefore, if the government can rationally plan the development of the pension industry, the "second demographic dividend" will hopefully drive economic growth again. Hongjun Qiu believes that by rationally integrating the resources of different regions and extending the industry chain of tourism for the elderly, not only can it effectively alleviate the expansion of the urban population, but also promote the economic development of small and medium-sized cities [27]. In addition to focusing on the characteristics of the elderly who have relatively more savings, the educational and academic background of the elderly is also worth analyzing. Individuals with strong academic backgrounds or high levels of education who are newly entering old age can positively influence the development of the regional economy [28] and can partially mitigate the negative effects of the overall elderly population [1]. In addition to the above factors, Khan Mamun [29] and Galappaththi Kethaka [30] have proved that there is a positive correlation between population aging and economic growth. Meanwhile, with the further deepening of research on population aging by scholars around the world, many scholars believe that the "silver hair" economy will help promote economic development [31,32]. The impacts of population aging are not all negative [33,34].

In the third view, some scholars believe that the impact of population aging on economic growth needs further research. These scholars argue that the impact of population aging on the economy may depend on national policies [35,36], the supply of human capital [37], and even the extent of hospital care input [38] and other reasons. Therefore, they believe that the impact of population aging on economic development is not a simple relationship of growth or suppression [39].

Based on this, the first hypothesis is formulated:

H1: Population aging has a significant impact on economic development.

### 2.2 Health expenditures and economic development

In the process of economic development, human capital is one of the important influencing factors, and health expenditure is an important prerequisite for the development of human capital. In the existing literature, there is no uniform and definitive conclusion on the relationship between health expenditure and economic development, and there are three main views:

First, scholars have argued that health spending improves the health of the population, which in turn affects the share of the labor force in the demographic structure, and that an increase in the share of the labor force helps to promote economic development [40]. Analyzed from another perspective, the increase in the share of government health expenditure contributes to the reduction of personal health expenditure, which in turn improves personal income. According to the results of Bhargava’s study, it can be seen that there is a positive correlation between income and health [41]. Apart from the fact that higher government expenditure on health contributes to economic growth, there is also an impact of social and personal expenditure on health on economic development. Guisan argues that increased public and personal expenditure on health increases individual well-being [42]. At the same time, the health expenditure perspective suggests that there is a long-term correlation between health expenditure and economic development [43,44]. In addition to this, it has also been argued that higher health expenditure helps to boost personal output and hence personal GDP, thus impacting economic development [45].

Second, some scholars argue that while higher health spending can help improve individual health, it can place a burden on economic growth [46,47]. The literature in this category is relatively small and the references are dated. Finally, some scholars have argued that the relationship between health spending and economic development is not simply linear [48]. Moreover, the impact of health expenditures on economic development may have different results depending on the economic development of the region [49].

Based on this, a second hypothesis is proposed:

H2: Health expenditure has a significant impact on economic development.

In summary, although a large number of domestic and foreign literature on the relationship between population aging and economic development has conducted a large number of in-depth studies, the conclusions of the studies have not reached effective unity, and in the existing research, most of the studies on population aging take into account the direct and indirect impacts of population aging on the economy and do not take into account the elderly population’s factors, such as the elderly health expenditures, education level. At the same time, most of the current literature is to study the unilateral impact of population aging on the economy, and few scholars have included population aging, health expenditure, education of the elderly, urbanization level, and economic development in the same research framework for empirical analysis. At the same time, this paper distinguishes health expenditure as government health expenditure, social health expenditure, and personal health expenditure, and analyzes the impact of health expenditure on economic development for population aging from three perspectives. Based on this, this paper empirically examines the impact of population aging on economic development from the perspective of health investment based on the control panel model and DIFF-GMM model using the provincial panel data of 30 provinces, municipalities directly under the central government and autonomous regions of China (excluding Tibet, Hong Kong, Macao, and Taiwan) for the period of 2000–2021 against the background of big data and draws the corresponding conclusions.

## 3. Research design

### 3.1 Data sources and variable definitions

#### 3.1.1 Data sources

This study is based on provincial-level panel data from 30 Chinese provinces (municipalities and autonomous regions) from 2000–2021, excluding Tibet, Hong Kong, Macao, and Taiwan for reasons of data availability and completeness. The data for each variable are obtained from the China Statistical Yearbook, the Statistical Yearbook of each province, the China Population and Employment Statistical Yearbook, the China Health and Sanitation Statistical Yearbook, and the relevant reports published by the National Bureau of Statistics and the Finance Bureau of each province from 2001 to 2022. At the same time, to avoid the influence of the price factor, some variables are deflated with 2000 as the base period.

#### 3.1.2 Definition of variables

1. Explained variables

The explanatory variable in this paper is the Gross Domestic Product (GDP).

2. Core explanatory variables

Population aging (AGING), this paper uses the ratio of the population aged 65 and above to the total population as the explanatory variable in the empirical test section; and replaces the ratio of the population aged 65 and above to the total population with the dependency ratio of the elderly population (ODR) in the robustness test section. Health Expenditures (HE), are measured by individual health expenditures, social health expenditures, and government health expenditures respectively.

3. Underlying explanatory variables

Capital input (K), measured using logarithmized gross fixed capital. The level of labor supply (LAB), referring to the research design of Jianfeng Feng and Weimin Chen, is measured using the product of the number of years of schooling and the number of employed persons per capita in the region [50]. The level of urbanization (CITY), is measured using the proportion of the regional urban population to the total regional population. Population Growth Level (PGR), which introduces the regional population growth rate into the model. The level of trade openness (OPEN), is measured using the ratio of total regional imports and exports to regional GDP.

Variable definitions and variable descriptive statistics are specified in Tables 1 and 2.

**Table 1:** Definition of variables.

Variable category	variable name	variable symbol	Variable Definition
**explanatory variable**	economic development	GDP	Gross domestic product (billions of dollars)
**explanatory variable**	population aging	AGING	In the empirical section, the ratio of the population aged 65 and over to the total population is used as the explanatory variable; in the robustness test section, the ratio of the population aged 65 and over to the total population is replaced by the dependency ratio of the elderly population.
	Health expenditure	HE	Measured as population health expenditure (HHE), social health expenditure (SHE), and government health expenditure (GHE), respectively
**control variable**	capital investment	K	Total fixed capital (billions of dollars)
	Level of labor supply	LAB	Natural logarithm of the product of years of schooling and employment per capita
	urbanization level (of a city or town)	CITY	Regional urbanization rate
	Level of population growth	PGR	population growth rate
	Level of trade openness	OPEN	Total exports and imports as a percentage of GDP

**Table 2:** Descriptive statistics of variables.

variant		sample size	average value	(statistics) standard deviation	minimum value	maximum values
GDP		660	4115.781	3335.213	117.464	15082.175
AGING	POP65	660	10.874	2.582	4.257	19.872
	ODR	660	14.767	3.159	4.331	24.892
HE	HHE	660	771.138	212.845	200.121	1487.593
	SHE	660	37.182	8.218	18.401	61.182
	GHE	660	31.862	7.256	60.376	17.887
K		660	4818.903	4668.211	38.264	26885.337
LAB		660	9.530	0.732	7.515	11.011
CITY		660	60.127	13.091	14.29	89.300
PGR		660	5.171	3.135	-5.8	13.10
OPEN		660	32.001	37.672	1.105	178.987

### 3.2 Modeling

This study refers to the research design of Lv Guoying [51]. The research design of this study adopts the basic form of the Cobb-Douglas production function (C-D production function) (Y = A(_t_)LK_α_β_μ_) as the model basis, and to investigate the impact of population aging and health expenditure on economic development, this study firstly introduces population aging and health expenditure into the Cobb-Douglas production function at the same time to establish a basic panel model. Secondly, in order to strengthen the regression effect of the model, control variables are added to the panel model to control the impact of the heterogeneity of various variables in the regression on economic development. Then, to reduce the negative impact of heteroskedasticity and covariance on the empirical test, the natural logarithm is taken to make the study more rigorous; at the same time, to reduce the impact of the endogeneity problem caused by the laggedness of the economic development and the reverse causality between the variables on the empirical test, the lagged period of the explanatory variables is incorporated into the model in this study, and a dynamic panel model is established (1). In order to explore the interaction between population aging and health expenditure on economic development, the interaction term between population aging and health expenditure is introduced into the dynamic panel model (1) to establish a new dynamic panel model (2) as follows:

$$\begin{array}{l} Ln\;GDP_{it}=\alpha +\beta_{0}\ln\;GDP_{i,\;t‐1}+\beta_{1}\ln\;AGING_{it}+\beta_{2}\ln\;HE_{it}\\ \;+\beta_{3}\ln\;K_{it}+\beta_{4}\ln\;LAB_{it}+\beta_{5}\ln\;CITY_{it}+\beta_{6}\ln\;PGR_{it}\\ \;+\beta_{7}\ln\;OPEN_{it}+\varepsilon_{it} \end{array}$$ (1)

$$\begin{array}{l} Ln\;GDP_{it}\;=\alpha +\beta_{0}\ln\;GDP_{i,\;t‐1}+\beta_{1}\ln\;AGING_{it}+\beta_{2}\ln\;HE_{it}\\ \;+\beta_{3}\ln\;K_{it}+\beta_{4}\ln\;LAB_{it}+\beta_{5}\ln\;CITY_{it}\;+\beta_{6}\ln\;PGR_{it}\\ \;+\beta_{7}\ln\;OPEN_{it}\;+\beta_{8}\ln\;H_{it}A_{it}\;+\varepsilon_{it}\; \end{array}$$ (2)

Where α is a constant term, β_0_-β_9_ represent the coefficients of each variable, i and t represent province and time, respectively, μ_i_, and v_t_ represent area fixed effects and year fixed effects, respectively, and ε_it_ is the residual.

### 3.3 Choice of empirical methodology

To avoid the problem of endogeneity of the model on the empirical test of the results of the bias problem, this study in the empirical test part of the model uses the DIFF-GMM method to analyze the model. Meanwhile, to enhance the validity of the empirical results, this study sets the fixed effect model (FE) and fixed effect instrumental variable model (FE-IV) as a reference. To test the validity of the model, the Hanser test and AR test are used.

## 4. Empirical results and analysis

### 4.1 Benchmark regression results

The results of the empirical tests are shown in Table 3. According to the results of the benchmark regression in Table 3, it can be seen that the P-value of AR (1) is lower than 0.1; the P-value of both AR (2) and H-test is greater than 0.1, which proves the validity of the instrumental variables used in the empirical process. The indicator used in the empirical evidence to measure the degree of aging of the society—the share of the population aged 65 and above in the total population (POP65) has a significant inhibitory effect on economic development, all of which are significant at the 99% level confidence interval, a finding that validates the correctness of H1. Meanwhile, the three indicators representing health expenditure (HE): personal health expenditure (HHE), social health expenditure (SHE), and government health expenditure (GHE) all passed the 1% significance level test, indicating that the health expenditure brought about a promotion effect on economic development, and this conclusion verifies the correctness of H2. The empirical results show that the deepening of population aging will hurt economic development, while the growth of health expenditures (whether personal health expenditures, social health expenditures, or government health expenditures) has a positive effect on economic development. In addition to the indicators of population aging and health expenditures, fixed capital investment (K), the number of years of education and employment per capita (LAB), and the level of urbanization (CITY) all have a positive impact on economic development, indicating that an increase in capital investment, labor investment, years of education per capita, and an increase in China’s level of urbanization can promote the further development of China’s economy, which is in line with the reality of China’s economic development. This result is consistent with the reality of China’s economic development. According to the theory of economic growth, an increase in the input level of capital and labor factors will promote economic growth. An increase in the number of years of education per capita means an increase in the state’s investment in education, which will have an important impact on the increase of human capital factors, and the increase of human capital factors can also drive economic development. At the same time, the increase in urbanization level brings significant promotion effect on economic development, the specific reason can be attributed to the urbanization brings capital pooling, labor concentration, transportation, and education radiation enhancement, science, technology, information development, and other factors, will show a certain degree of promotion effect on the economy. In addition, the increase in the level of population growth (PGR) and the level of trade openness (OPEN) hurt the economy, but the regression results are not significant.

**Table 3:** Empirical results of population aging, health expenditure on economic development.

explanatory variable	HHE			SHE			GHE		
	DIFF-GMM	FE	FE-IV	DIFF-GMM	FE	FE-IV	DIFF-GMM	FE	FE-IV
GDP	0.479*** (0.09)			0.481*** (0.10)			0.453*** (0.08)		
POP_65_	-0.144*** (0.03)	-0.061*** (0.02)	-0.138*** (0.02)	-0.149*** (0.05)	-0.062*** (0.02)	-0.151*** (0.05)	-0.145*** (0.03)	-0.039*** (0.02)	-0.105*** (0.02)
HHE	0.091*** (0.03)	0.102*** (0.03)	0.145*** (0.03)						
SHE				0.098*** (0.02)	0.105*** (0.03)	0.155*** (0.05)			
GHE							0.054*** (0.01)	0.077*** (0.02)	0.097*** (0.02)
K	0.029*** (0.01)	0.066*** (0.01)	0.063*** (0.01)	0.033*** (0.01)	0.069*** (0.01)	0.067*** (0.01)	0.020** (0.01)	0.045*** (0.01)	0.047*** (0.01)
LAB	0.228* (0.15)	0.359*** (0.06)	0.306*** (0.06)	0.235 (0.16)	0.365*** (0.06)	0.311*** (0.06)	0.121 (0.11)	0.270*** (0.05)	0.168*** (0.04)
CITY	0.047 (0.05)	0.079*** (0.03)	0.065** (0.03)	0.051 (0.04)	0.087* (0.05)	0.075* (0.04)	0.065** (0.03)	0.074*** (0.02)	0.076*** (0.02)
PGR	-0.003 (0.01)	0.019* (0.01)	0.012 (0.01)	-0.005 (0.01)	0.020* (0.01)	0.014 (0.01)	-0.009 (0.01)	0.016* (0.01)	0.008 (0.01)
OPEN	-0.013 (0.03)	-0.011 (0.01)	-0.004 (0.01)	0.013 (0.02)	-0.020* (0.01)	-0.013 (0.01)	0.011 (0.02)	-0.018* (0.01)	-0.012 (0.01)
constant term (math.)		7.216*** (0.33)	7.721*** (0.34)		0.733*** (0.35)	0.782*** (0.35)		0.748*** (0.29)	0.795*** (0.31)
R^2^		0.919	0.920		0.923	0.927			
AR(1)	0.001			0.001			0.000		
AR(2)	0.257			0.261			0.223		
Hansen	0.551			0.592			0.589		
N	600	660	660	600	660	660	600	660	660

Note: Standard errors are in parentheses; ***, **, and * represent 1%, 5% & 10% significance levels, respectively.

### 4.2 Analysis of regression results of the interaction between population aging and health expenditure

In the benchmark regression, the effects of population aging and health expenditure on economic development are empirically tested, and the next step is to empirically test the interaction between population aging and health expenditure on economic development, still using the DIFF-GMM method to analyze the model (2), and also setting up a fixed-effects model (FE) and a fixed-effects instrumental variables model (FE-IV) as a reference, and the results of the regression are shown in Table 4. If the regression coefficients of the interaction term of population aging and health expenditure are positive, it means that population aging and health expenditure have a positive contribution to economic development; on the contrary, it shows an inhibitory effect.

**Table 4:** Interaction between population aging and health expenditure on economic development.

explanatory variable	HHE			SHE			GHE		
	DIFF-GMM	FE	FE-IV	DIFF-GMM	FE	FE-IV	DIFF-GMM	FE	FE-IV
GDP	0.338*** (0.13)			0.355*** (0.11)			0.351*** (0.11)		
HHE-POP_65_	-0.057* (0.03)	-0.061*** (0.01)	-0.219*** (0.06)						
SHE-POP_65_				-0.060* (0.03)	-0.066*** (0.01)	-0.222*** (0.04)			
GHE-POP_65_							-0.055* (0.03)	-0.065*** (0.01)	-0.221*** (0.04)
POP_65_	0.141 (0.15)	0.201*** (0.04)	0.839*** (0.14)	0.162 (0.15)	0.205*** (0.03)	0.897*** (0.25)	0.160 (0.15)	0.192*** (0.03)	0.891*** (0.27)
HHE	0.225** (0.11)	0.283*** (0.04)	0.701*** (0.15)						
SHE				0.231* (0.13)	0.289*** (0.04))	0.717*** (0.12)			
GHE							0.226** (0.10)	0.288*** (0.04)	0.706*** (0.12)
K	0.038* (0.02)	0.053*** (0.01)	0.045*** (0.01)	0.040* (0.02)	0.055*** (0.01)	0.048*** (0.01)	0.021** (0.01)	0.035*** (0.01)	-0.003 (0.01)
LAB	0.166* (0.11)	0.291*** (0.05)	0.276*** (0.05)	0.175* (0.11)	0.295*** (0.05)	0.284*** (0.06)	0.110 (0.09)	0.192*** (0.04)	0.128*** (0.04)
CITY	0.058* (0.03)	0.073*** (0.02)	0.025 (0.03)	0.091*** (0.03)	0.101*** (0.02)	0.065*** (0.02)	0.090*** (0.03)	0.098*** (0.02)	0.059*** (0.02)
PGR	0.011 (0.05)	0.022** (0.01)	0.023** (0.01)	0.012 (0.05)	0.030*** (0.01)	0.035*** (0.01)	0.007 (0.01)	0.029*** (0.01)	0.033*** (0.01)
OPEN	0.035* (0.02)	-0.007 (0.01)	0.023** (0.01)	0.025** (0.01)	-0.012 (0.01)	0.019* (0.01)	0.012 (0.03)	-0.015 (0.01)	0.014 (0.01)
constant term (math.)		6.883*** (0.31)	5.992*** (0.43)					7.335*** (0.28)	7.175*** (0.30)
R^2^		0.921	0.911					0.940	0.928
AR(1)	0.003			0.002			0.002		
AR(2)	0.070			0.071			0.284		
Hansen	0.751			0.755			0.190		
N	600	660	660	600	660	660	600	660	660

According to the estimation results in Table 4, it can be seen that the coefficients of the interaction term between population aging and personal health expenditure (HHE-POP65), social health expenditure (SHE-POP65), and governmental health expenditure (GHE-POP65) are -0.051, -0.060, and -0.055, respectively, which all pass the test of the level of significance, which results in proving that either personal health expenditure or social health expenditure or government health expenditure on economic development and the impact of population aging on economic development is a mutually inhibiting relationship. The specific effect is that if the individual, social, or government health expenditure increases, the inhibitory effect of population aging on the economy will be effectively alleviated; similarly, as the degree of social aging deepens, the promotion effect of health expenditure on economic development will be weakened as a result. The results obtained by the fixed-effects model and the fixed-effects instrumental variables model are similar to those of the DIFF-GMM estimation.

### 4.3 Robustness tests

Although this study empirically tests the effects of population aging and health expenditure on economic development alone and the interaction of population aging and health expenditure on economic development, to improve the reliability of the empirical evidence, this part will be a robustness test of the effects of population aging and health expenditure on economic development. To avoid statistical errors and sample selection errors on the empirical evidence, the first part of the robustness test is the robustness test of the replacement variable, which is to replace the GDP indicator with the nighttime lighting data (DN), which is also one of the most important indicators reflecting the regional economic situation, according to Corning Xu [52]. Secondly, the elderly population dependency ratio (ODR) is used to replace the proportion of the population aged 65 and above in the total population (POP_65_), the elderly population dependency ratio represents the proportion of the elderly population to the labor force, and changes in this ratio can reflect the changes in the labor force and the aging population. The second part of the robustness test is the robustness test excluding some special sample data. Since municipalities are under the direct jurisdiction of the central government, they have a special status, and compared with ordinary provinces, they have obvious advantages in population concentration, at the same time, municipalities also have an important status in economy, politics, culture, etc. Therefore, to avoid the interference of the municipalities’ related data on the test, we chose to exclude them from the sample to carry out the robustness test. The third part of the robustness test is to divide the sample data into 2000–2010 2011–2021. China’s rapid economic development, population mobility, and technological development of China in different periods also have differences, so to exclude some of the special samples on the overall empirical results of the bias, the total sample is divided into the 2000–2010 and 2011–2021 time period, to carry out the robustness test.

#### 4.3.1 Robustness tests for replacement variables

Table 5 shows the results of the DIFF-GMM robustness test for replacing the GDP indicator with nighttime light data and using the elderly population dependency ratio to replace the share of the population aged 65 and above in the total population. According to the robustness test in Table 5, it can be seen that the independent effects and interactions of population aging and health expenditures passed the Hansen test and AR test. From the results, it can be seen that after replacing the variables, the dependency ratio of the elderly population still has a significant inhibitory effect on economic development; personal health expenditure, social health expenditure, and government health expenditure can promote economic growth to varying degrees; and the coefficient of the interaction term between the dependency ratio of the elderly population and the health expenditure is also negative, which is in line with the estimation results in Table 4, which means that the empirical results are not changed by replacing the indexes to change, representing that the empirical results are robust.

**Table 5:** Robustness test results for replacement variables.

explanatory variable	Independent influence			interaction		
	HHE	SHE	GHE	HHE	SHE	GHE
DN	0.485*** (0.10)	0.490*** (0.11)	0.233*** (0.08)	0.268*** (0.07)	0.478*** (0.10)	0.329*** (0.12)
HHE-ODR				-0.059** (0.03)		
SHE-ODR					-0.075** (0.03)	
GHE-ODR						-0.071** (0.03)
ODR	-0.166*** (0.04)	-0.170*** (0.04)	-0.073** (0.03)	0.242* (0.15)	0.253* (0.15)	0.132 (0.12)
HHE	0.081** (0.03)			0.255*** (0.10)		
SHE		0.103*** (0.01)			0.277*** (0.10)	
GHE			0.095*** (0.01)			0.218*** (0.07)
AR(1)	0.001	0.029	0.027	0.008	0.010	0.002
AR(2)	0.241	0.289	0.538	0.081	0.395	0.378
Hansen	0.534	0.611	0.199	0.146	0.181	0.170
N	600	600	600	600	600	600

#### 4.3.2 Robustness tests for excluding exceptional samples

Table 6 shows the results of the DIFF-GMM robustness test for the sample data excluding special municipalities. From the estimation results in Table 6, it can be seen that both independent effects and interactions are consistent with the previous results. Therefore, from the results of the robustness test with the municipality sample excluded, it can be seen that the empirical results have not changed because of the exclusion of the municipality sample, and the coefficients of the interaction terms of the inhibitory effect of population aging on the economy, the promotional effect of health expenditures on the economy, or the interaction effects are still negative, which indicates that the results of the empirical test are robust.

**Table 6:** Robustness test results excluding exceptional data.

explanatory variable	Independent influence			interaction		
	HHE	SHE	GHE	HHE	SHE	GHE
GDP	0.468*** (0.09)	0.480*** (0.10)	0.447*** (0.08)	0.324*** (0.11)	0.349*** (0.10)	0.345*** (0.10)
HHE-POP_65_				-0.053** (0.03)		
SHE-POP_65_					-0.070** (0.03)	
GHE-POP_65_						-0.063** (0.03)
POP_65_	-0.158*** (0.04)	-0.164*** (0.04)	-0.066** (0.03)	0.231* (0.14)	0.245* (0.15)	0.123 (0.11)
HHE	0.071** (0.03)			0.248*** (0.09)		
SHE		0.096*** (0.01)			0.271*** (0.09)	
GHE			0.091*** (0.01)			0.209*** (0.06)
AR(1)	0.001	0.030	0.029	0.007	0.011	0.002
AR(2)	0.262	0.298	0.545	0.092	0.389	0.382
Hansen	0.612	0.625	0.218	0.162	0.191	0.188
N	520	520	520	520	520	520

#### 4.3.3 Robustness tests for delineation time

Tables 7 and 8 show the results of DIFF-GMM robustness tests for 2000–2010 and 2011–2021, respectively. As can be seen from Tables 7 and 8, China has entered an aging society since 2000, and as the degree of aging deepens, the dampening effect on the economy also intensifies. Meanwhile, according to the robust results, it can be seen that from the period of 2000–2021, both personal health expenditure, social health expenditure, and government health expenditure have a significant role in promoting economic development. In addition to this, the interactions in Tables 7 and 8 show that the increase in health expenditures in these two time periods helps to mitigate the dampening effect of population aging on economic development. Similarly, it can be seen that the deepening of population aging can weaken the promoting effect of health expenditure on economic development. Based on the results of the robustness test for the periods, it can be seen that the empirical findings of this study are highly reliable and can pass the robustness test.

**Table 7:** Robustness test results for 2000–2010.

explanatory variable	Independent influence			interaction		
	HHE	SHE	GHE	HHE	SHE	GHE
GDP	0.461*** (0.06)	0.492*** (0.06)	0.467*** (0.15)	0.368*** (0.04)	0.349*** (0.10)	0.334*** (0.10)
HHE-POP_65_				-0.069** (0.03)		
SHE-POP_65_					-0.080** (0.03)	
GHE-POP_65_						-0.079** (0.04)
POP_65_	-0.119** (0.04)	-0.095** (0.04)	-0.078*** (0.03)	0.343 (0.33)	0.358 (0.33)	0.305 (0.31)
HHE	0.189*** (0.06)			0.318*** (0.09)		
SHE		0.96*** (0.01)			0.323*** (0.09)	
GHE			0.218*** (0.013)			0.178** (0.09)
AR(1)	0.022	0.023	0.003	0.062	0.063	0.062
AR(2)	0.568	0.575	0.172	0.259	0.274	0.182
Hansen	0.556	0.611	0.592	0.346	0.357	0.316
N	300	300	300	300	300	300

**Table 8:** Robustness test results for 2011–2021 data.

explanatory variable	Independent influence			interaction		
	
	HHE	SHE	GHE	HHE	SHE	GHE
GDP	0.462** (0.22)	0.495*** (0.14)	0.483*** (0.14)	0.565*** (0.11)	0.583*** (0.11)	0.571*** (0.11)
HHE-POP_65_				-0.055* (0.03)		
SHE-POP_65_					-0.082** (0.03)	
GHE-POP_65_						-0.080** (0.03)
POP_65_	-0.169* (0.09)	-0.176* (0.09)	-0.157** (0.08)	-0.258* (0.15)	-0.309** (0.15)	-0.379** (0.19)
HHE	0.082*** (0.03)			-0.176*** (0.06)		
SHE		0.096*** (0.04)			-0.286*** (0.09)	
GHE			0.077*** (0.02)			-0.278*** (0.09)
AR(1)	0.011	0.012	0.009	0.029	0.021	0.001
AR(2)	0.195	0.332	0.311	0.204	0.339	0.325
Hansen	0.574	0.695	0.649	0.986	0.989	0.633
N	300	300	300	300	300	300

## 5. Conclusions and recommendations

### 5.1 Conclusion

Based on the provincial panel data of 30 provinces (municipalities and autonomous regions) in China from 2000 to 2021, this study empirically investigates the relationship between population aging, health expenditures, and economic development with the DIFF-GMM estimation model, the fixed-effects model (FE) and the fixed-effects instrumental variables model (FE-IV). The following two conclusions are drawn from the empirical study: (1) Population aging has a significant inhibitory effect on economic development, and health expenditures of individuals, society, and the government can have a positive impact on economic development. (2) In further research, it is found that the increase in individual health expenditure, social health expenditure, and government health expenditure can help alleviate the negative impact of population aging on economic development. However, at the same time, the deepening of population aging will likewise inhibit the positive effect of health expenditure on economic growth. The results of the above empirical tests have passed the robustness tests of replacing key samples, excluding municipality samples and periods, and the results still hold.

### 5.2 Policy recommendations

Based on the above empirical findings, the following recommendations are made by both the government and society, as well as individuals.

From the perspective of the government and society, the government and society should continue to expand the scale of financial investment in the field of health, and give full play to the role of government and social health expenditures; an increase in the proportion of government and social health expenditures to total health expenditures will help to reduce personal health expenditures, and reduce the burden of medical care on the elderly, as well as the problems of "difficult to see" and "expensive to see" will be solved. The solution to the problem of "difficult" and "expensive" medical care can promote the successful realization of the strategic goal of "Healthy China". Specific practices are as follows: First, we can improve China’s health service system for the elderly, build a pension service system that combines "medical care" and "health care" for the elderly, promote the proper allocation of medical institutions and medical personnel, and build a specialized health service team for the elderly, to improve the health of the elderly in the new economic development. The Government is also promoting the proper allocation of medical institutions and medical personnel, and the establishment of specialized health service teams for the elderly, to meet the health requirements of the elderly with different service needs in the context of the new economic development. Second, based on the existing medical insurance system, the China Healthcare Commission should take the lead in joining forces with the departments of human Resources and social Welfare, Civil Affairs, and insurance to introduce a long-term care system and build a health insurance system for the elderly. Specifically, we can introduce the system of private doctors in the homes of developed countries such as Europe, the United States, and Japan, build a multidisciplinary collaborative diagnosis and treatment platform for the elderly with regard to chronic diseases, and promote an increase in the number of nursing staff in specialized nursing and rehabilitation institutions, so as to promote the participation of nursing through out the entire process of care from health care, prevention, treatment and rehabilitation, and to raise the level of nursing care for the elderly and the strength of its provision from the perspective of health care institutions. From the perspective of medical insurance for the elderly, set up medical insurance or accident insurance specifically for the health of the elderly, with insurance as a third-party supplement to reduce the medical expenses of the elderly due to medical services, and increase the savings of residents’ families. A perfect care system and health insurance system can not only reduce the time and economic pressure brought by the labor force to take care of the disabled and semi-disabled elderly but also release the labor force, and increase labor force participation and productivity, thus promoting economic development. Third, accelerate the development of aging-appropriate technologies and products, cultivate intelligent aging, promote social aging and other new forms of business, and give full play to the role of the "silver-hair economy", so as to alleviate the negative impacts of population aging on China’s industrial and economic development, and promote economic development.

From an individual perspective, it is important to build a healthcare system for the elderly population and to promote the healthy development of the healthcare industry for the elderly. There are two specific approaches: the first is to strengthen health education for the elderly using health lectures for the elderly jointly conducted by the Health and Welfare Commission, the Ministry of Education, the General Administration of Physical Education, and other relevant departments, as well as using senior citizens’ colleges and universities, health-knowledge campaigns, and the promotion of exercise for the elderly, in order to raise the health awareness of the entire population; this heightened awareness of health care will help the elderly to take the initiative in paying attention to their own health, thereby preventing disease and promoting the "healthization" of the elderly population. Second, promote the regularization of medical check-ups for the elderly population. Second, promote the regularization of medical checkups for the elderly population. Local governments and hospitals should take the lead in reasonably guiding and encouraging the elderly to undergo medical checkups on a quarterly or half-yearly basis; regularization of medical checkups can help detect early diseases, monitor the development of chronic diseases, and adjust the state of subhealth.

From the perspectives of the Government, society, and individuals, reasonable means and measures should be adopted to maximize the life expectancy of the elderly population, reasonably safeguard the efficiency of the use of the human capital of the elderly, reduce the burden on the family and society, and promote the development of the "silver-hair economy", to promote the benign development of the economy.

## 6. Analysis of limitations

Although this study has some innovations based on the existing literature, it still has limitations. First, although China is the largest developing country in the world, the development history of China’s economy is unique, and the impact of China’s population aging on economic development may be able to give lessons to other developing countries, but more data samples are needed, and future research can incorporate demographic data from more developing countries into the empirical study. Second, there are still deficiencies in the selection of variables for empirical research. Economic development is a dynamic and complex process, and although variables such as the level of labor supply, the level of population growth, and the level of urbanization are selected in the study, they may not be able to include all the factors that affect economic development. Therefore, in the next step of the study, more variables affecting economic development may be included in the research framework. Both of these shortcomings will be something that future research will need to overcome.

## Supporting information

S1 Appendix Data. (XLSX)
